# Polarized proton spin density images the tyrosyl radical locations in bovine liver catalase

**DOI:** 10.1107/S205225251601054X

**Published:** 2016-07-29

**Authors:** Oliver Zimmer, Hélène M. Jouve, Heinrich B. Stuhrmann

**Affiliations:** a Institut Laue-Langevin, F-38042 Grenoble, CEDEX 9, France; b Institut de Biologie Structurale Jean-Pierre Ebel, CEA/CNRS/UJF, F-38027 Grenoble, CEDEX 1, France; c Helmholtz-Zentrum Geesthacht, D-21494 Geesthacht, Germany

**Keywords:** polarized neutron scattering, dynamic nuclear polarization, magnetic nuclear spin diffusion barrier, tyrosyl in catalase

## Abstract

A tyrosyl radical, as part of the amino acid chain of bovine liver catalase, supports dynamic proton spin polarization (DNP). Finding the position of the tyrosyl radical within the macromolecule relies on the accumulation of proton polarization close to it, which is readily observed by polarized neutron scattering. The nuclear scattering amplitude due to the polarization of protons less than 10 Å distant from the tyrosyl radical is ten times larger than the amplitude of magnetic neutron scattering from an unpaired polarized electron of the same radical. The direction of DNP was inverted every 5 s, and the initial evolution of the intensity of polarized neutron scattering after each inversion was used to identify those tyrosines which have assumed a radical state. Three radical sites, all of them close to the molecular centre and the haem, appear to be equally possible. Among these is tyr-369, the radical state of which had previously been proven by electron paramagnetic resonance.

## Abstract 2

Radical sites in catalase have been located by the method of time-resolved polarized neutron scattering from dynamically polarized protons. This method is about ten times more sensitive than magnetic neutron scattering and therefore particularly suitable for very dilute paramagnets.

## 1. Introduction   

At first glance, it appears that an enzyme like catalase, even if radical-doped, does not have much in common with polarized proton spin targets. The latter are used in experiments in high-energy physics, whereas a redox enzyme like catalase is involved in the life sciences. We will show that it is polarized neutron scattering which connects the two worlds. Before entering into this complex undertaking, it seems appropriate to describe some aspects of both worlds which may bear a tremendous potential for synergies in neutron scattering.

Let us start with dynamically polarized protons. From NMR spectroscopy, it is well established that protons in organic radical molecules reach thermal equilibrium with the lattice *via* interaction with their unpaired electrons. The principle of this nuclear relaxation process states that proton spins near unpaired electrons are relaxed by fluctuations in the magnetic field of the unpaired electron (direct relaxation) and that bulk proton spins are relaxed by diffusion of Zeeman energy to neighbouring protons (spin diffusion) (Bloembergen, 1949 ▸). The number of close protons in direct contact with the non-Zeeman electron-spin–electron-spin interaction reservoir (or bath) is limited by the fact that the local spin lattice relaxation rate is inversely proportional to the sixth power of the distance between the unpaired electron and the proton (Wolfe, 1973 ▸; Furman & Goren, 2002 ▸). The result is a gradient of nuclear polarization reflecting a nuclear spin diffusion barrier which separates the close nuclei subject to fast relaxation from those of the bulk. As the local magnetic field near an unpaired electron is very large, the Larmor frequencies of the close nuclei differ appreciably from those of the bulk. Consequently, the close nuclei hardly contribute to the NMR signal from the bulk. It is this picture which provides the environment for dynamic nuclear spin polarization (DNP).

DNP is a widely used method to polarize nuclear spins. The macroscopic aspects of DNP are well understood within the framework of the spin temperature theory (Abragam & Goldman, 1978 ▸). The thermodynamic model of DNP assigns two heat reservoirs to various degrees of freedom of the electronic and nuclear spin systems that are coupled *via* mutual and external interactions (Abragam & Goldman, 1982 ▸). The mechanism of DNP is then described as a two-step process: the cooling of the electronic non-Zeeman reservoir by a non-saturating microwave field at frequencies close to the electronic resonance, and the subsequent transfer of entropy from the nuclear Zeeman system consisting of the close protons. Depending on the type and density of the unpaired electrons in the sample and the external conditions (temperature, applied magnetic field), DNP proceeds either by the so-called solid effect or by thermal mixing or by a combination of them (Abragam & Goldman, 1978 ▸; Wencke­bach, 2016 ▸, and references therein). Note that the exact type of process is not relevant for the evaluation of the experimental data. In any case, it is assumed that this transfer is efficient so that the spin temperatures of both systems become equal (Borghini, 1968 ▸; Niinikoski, 2013 ▸). The magnitude of the mixing is strongly influenced by the microscopic structure of the material and, in particular, the nuclear spins close to the unpaired electron (Cox *et al.*, 1977 ▸; Jannin *et al.*, 2012 ▸). The transfer of the polarization of the close protons to those of the bulk is controlled by the magnetic spin diffusion barrier, as mentioned above. Hence, the polarization of the close protons is expected to be different from those of the bulk, particularly at the onset of microwave irradiation.

Now let us turn to radical proteins. Radicals play an important role in living cells and radical intermediates allow enzymes to perform a wide variety of chemically challenging reactions. Catalase is a redox enzyme which converts hydrogen peroxide at a very fast diffusion-limited rate into water and molecular oxygen. By offering peroxyacetic acid, a derivative of hydrogen peroxide with more steric hindrance, to bovine liver catalase, the reaction differs in at least two points: first, the response is slow, and second, after some intermediate steps, one of its amino acids, a tyrosine, is converted into a tyrosyl radical. It is assumed that one and only one of the 20 tyrosines of each of the four subunits of catalase becomes a tyrosyl radical (Ivancich *et al.*, 1997 ▸).

Finding the exact location of a (tyrosyl) radical is an important step in understanding the mechanism of an enzyme. Electron paramagnetic resonance (EPR) is the method of choice for detecting a radical, and in favourable cases the site of the radical inside a protein molecule can be obtained from a detailed analysis of the EPR spectrum (Svistunenko & Cooper, 2004 ▸). A more general approach to the determination of the location of an unpaired electron is provided by magnetic neutron scattering. However, a radical-doped protein can be considered as an extremely dilute paramagnet, with a correspondingly low sensitivity to detecting the magnetic amplitude.

When it comes to the size of the polarization-dependent scattering length, then polarized nuclei, and certainly protons, are more rewarding targets. This was realised 40 years ago, when the dynamically polarized proton spins in an Nd^3+^-doped crystal of lanthanum magnesium nitrate were studied by polarized neutron scattering (Hayter *et al.*, 1974 ▸; Leslie *et al.*, 1980 ▸). It took another ten years before dynamically polarized protons in noncrystalline targets considerably widened the range of applications of neutron scattering (Knop *et al.*, 1986 ▸; Kohgi *et al.*, 1987 ▸; Glättli *et al.*, 1989 ▸). These and later experiments on nuclear spin contrast variation (*e.g.* Willumeit *et al.*, 1996 ▸; Kumada *et al.*, 2010 ▸; Noda *et al.*, 2013 ▸), which profited from a high proton polarization-dependent scattering amplitude, prepared the ground for a more detailed study of the build-up of proton polarization by polarized neutron scattering. The starting signal came from a problem in structural biology concerning the location of a tyrosyl radical in bovine liver catalase (Ivancich *et al.*, 1997 ▸). In an initial step, it led to a collaboration of groups at the IBS, Grenoble, France, the CEN, Grenoble, and the PSI, Villigen, Switzerland.

## 2. First experiments of DNP with the tyrosyl radical   

**Figure 1:**
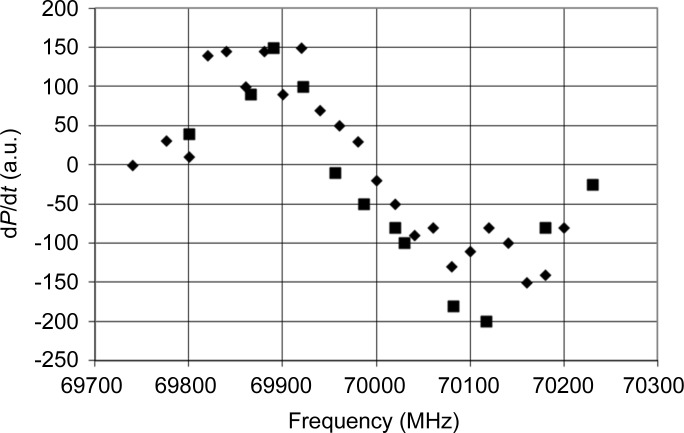
The direction and speed of dynamic proton polarization as a function of wavelength in the presence of free tyrosyl radicals (*c* = 10^18^ ml^−1^) in a glycerol–water mixture. The black squares and black diamonds indicate two different scans. *B* = 2.5 T.

In a preliminary study, the suitability of tyrosyl radicals for DNP was tested. For this purpose, a solution of tyrosine (20 m*M*) was irradiated by a UV lamp (240 W) for 15 min at liquid nitrogen temperature. The frozen sample was then put into an environment suitable for DNP: *T* = 1 K, *B* = 2.5 T. In a microwave frequency scan across the EPR line of the tyrosyl radical, an increase in the NMR intensity due to the bulk proton polarization *P* was observed at frequencies slightly below the resonance. At frequencies slightly above the EPR, *P* assumed negative values (Fig. 1 ▸). The unpaired electron of the tyrosyl does support DNP.

**Figure 2:**
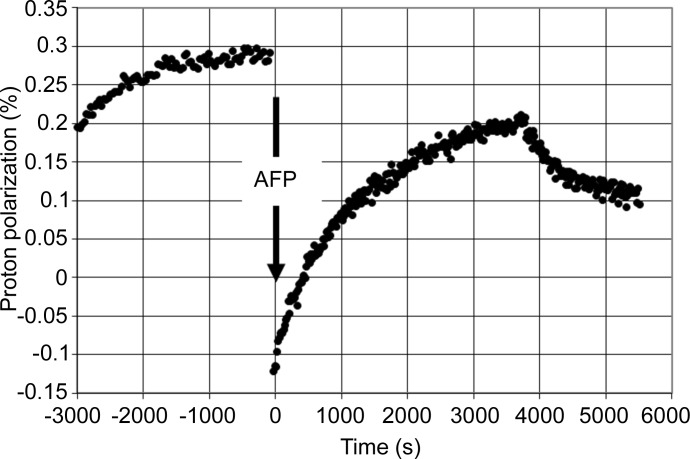
The NMR intensities from dynamically polarized protons of tyrosyl-doped catalase. *T* = 1 K and *B* = 2.5 T. At *t* = 0, the direction of proton polarization is reversed by the method of AFP. At *t* = 3800 s, the direction of DNP is reversed by a frequency jump.

This was good news for the next step, the dynamic polarization of protons in tyrosyl-doped catalase. The proton NMR intensity from dynamically polarized protons of catalase is shown in Fig. 2 ▸. After 1 h of microwave irradiation, the proton polarization *P* reaches a value of 0.3%, which is only slightly above the value of *P* at thermal equilibrium, *P*_e_ = 0.25% (*T* = 1 K and *B* = 2.5 T). A negative proton polarization of −0.12% is achieved by the method of adiabatic fast passage (AFP). Attempts to reach a negative proton polarization by dynamic polarization in the negative direction ended at *P* = 0.1% (Fig. 2 ▸). At *T* = 1 K, the average proton polarization thus remains close to *P*_e_, whatever direction of DNP is used.

From Fig. 2 ▸ we deduce two ways to create a non-equilibrium between close and bulk protons. The first and most visible one in this figure is the reversal of the polarization of the bulk protons by the method of AFP, and the second is the change in the direction of DNP which primarily affects the close protons. The method of AFP is very attractive from the theoretical point of view (Buckingham, 2003 ▸), but it bears several disadvantages. First, the efficiency of polarization reversal is low, and only one third of the initial polarization is left (Fig. 2 ▸). Moreover, the time needed to prepare an important polarization jump is long compared with the decay time of the polarization gradient, which results in a poor use of the neutron beam time. A solution to this problem is offered by frequently alternating the direction of dynamic polarization. The build-up of a polarization gradient is then observed after each change in direction of DNP, allowing for a high duty cycle. Another merit of this method is that it will focus on the polarization of the close protons, which are expected to image the electron spin density.

Another lesson drawn from Fig. 2 ▸ concerns the weakness of the proton polarization in catalase, which might seriously limit the information obtainable from neutron scattering. The experience gained from simpler systems could facilitate analysis of the data obtained with catalase. This input came from the group of Hans Glättli, CEN Saclay, France, proposing a study of the build-up of proton polarization in free radicals. In collaboration with the ILL and PSI, the protocol of time-resolved neutron scattering from dynamically polarized protons in solids was defined.

## 3. Experimental procedures   

A detailed description of the instruments and their use for time-resolved neutron scattering from dynamically polarized protons has been given earlier by van den Brandt *et al.* (2002 ▸, 2004 ▸, 2006 ▸). Here we present experimental details that are necessary in order to achieve a self-contained presentation.

### 3.1. Sample preparation   

The preparation of the sample started from tetramer bovine liver catalase (BLC) in crystalline suspension (Boehringer-Mannheim, Germany, lot 106 828). The suspension (12 ml) was dissolved in Tris maleate buffer (2 l; 50 m*M*, pH 7.5), purified, concentrated to 5 ml and dialyzed against 150 ml Tris maleate in D_2_O. The buffer was exchanged three times. The contents of the dialysis tube partly precipitated, but a clear solution (0.515 m*M* haem) was obtained by addition of an equal volume of deuterated glycerol. Note that the BLC molecule is a tetramer enzyme, with one haem as part of each of its four identical subunits. At a temperature only slightly above 0°C, to the clear solution of BLC (1 ml), peroxyacetic acid (Merck; 28 µl; 32% solution, diluted to a 3% solution in 0.1 *M* Tris, and adjusted to pH 4.3) was added. The immediate formation of a porphyrin-π-cation radical and the subsequent slower formation of a tyrosyl radical could be followed by a change of the colour to deep red. After a few minutes, the tyrosyl concentration reached its maximum and the sample was frozen to liquid nitrogen temperature. From the EPR measurements, a tyrosyl concentration of 0.58 per haem was determined (sample 1 in Table 1 ▸). Samples with a lower catalase concentration were prepared in the same way.

### 3.2. Small-angle neutron scattering (SANS)   

The SANS measurements were performed on the D22 instrument at the Institut Laue–Langevin (ILL), Grenoble. We used polarized incident neutrons of wavelength λ = 4.6 Å with a wavelength spread of δλ/λ = 0.1 (full width at half-maximum). The detector on D22 is a large-area single-vessel multiwire proportional counter using ^3^He gas at 1.85 bar (1 bar = 100 000 Pa) plus some CF_4_. It contains two orthogonal sets of 128 cathode wires and the position information is extracted by finding a coincidence of an *X* and a *Y* signal. Such a structure is limited to a counting rate of at most a few hundred kilohertz.

The solid sample was inserted into the NMR coil, placed in a microwave cavity and mounted inside a ^4^He refrigerator, which was operated at a temperature of about 1 K. A longitudinal static magnetic field of 3.5 T was provided by a superconducting split-coil solenoid. Samples could be changed and cooled down to 1 K in less than half an hour.

Two IMPATT diodes, tuned to frequencies required for positive and negative DNP, 97.20 and 97.50 GHz, respectively, could be connected to the sample cavity by a waveguide switch (total switching time 100 ms, comprised of shutting within 5 ms, no microwaves for 90 ms, opening in 5 ms). Continuous wave (cw) NMR was used in parallel to monitor the bulk proton polarization. The direction of DNP was reversed every 5 s, and the acquisition of the polarized neutron scattering intensity spectra was triggered synchronously at time intervals of 50 ms. Each of the 200 time frames was averaged over several thousand cycles to obtain sufficient statistical accuracy. The time-dependent scattering intensity was then obtained as usual by radial averaging, and corrected for polarization-dependent transmission and background scattering.

## 4. Mathematical formalism   

The evolution of proton polarization in space and time can be described in terms of rate equations that govern the flow of nuclear polarization between reservoirs coupled in series (Stuhrmann, 2004 ▸; van den Brandt *et al.*, 2006 ▸). The reservoirs can be identified as follows. As the source of DNP, we have the electronic spin–spin interaction reservoir R0. Depending on the choice of microwave frequency it will drive the nuclear polarization towards *P* = 1 or *P* = −1, *i.e.* reservoir R0 will assume the values *P*_0_ = ±1. R1 is the reservoir of close protons that are presumably inside a spherical magnetic spin diffusion barrier with a radius of 5 Å (see Appendix *A* [App appa]). In order to simulate a diffuse barrier, an additional reservoir, R2, of not-so-close protons inside a hollow sphere with an outer radius of *r*_2_ = 10 Å was assumed. R3 contains all the protons of the catalase molecule except those of R1 and R2. The protons of the deuterated solvent define R4. The protons in R3 and R4 are bulk protons, while those of R2 may be a mixture of close and bulk protons.

**Figure 3:**
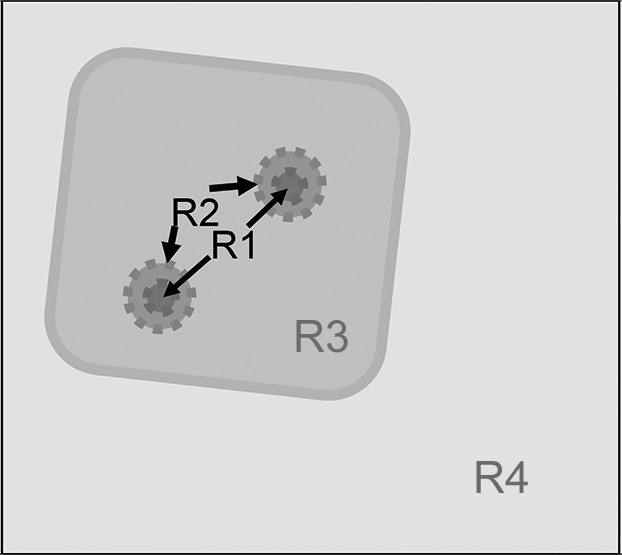
A schematic presentation of the catalase molecule, with its reservoirs R1, R2 and R3 embedded in a matrix R4. Close and not-so-close protons are contained in reservoirs R1 and R2, respectively. Protons of catalase outside the magnetic diffusion barrier (extending between the dashed circles) are found in R3. The efficiency of the isotopic diffusion barrier (grey line) increases with the deuteration of the matrix.

Assuming a spatial arrangement of the tyrosyls inside the catalase molecule as presented in Fig. 3 ▸, the build-up of proton polarization *P*_1_(*t*) in R1, *P*_2_(*t*) in R2, *P*_3_(*t*) in R3 and *P*_4_(*t*) in R4 is governed by a set four coupled first-order differential equations 
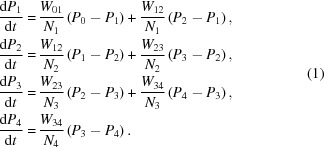
For the catalase molecule itself, *N_i_* is the number of protons in R*i* and *N*_4_ is the number of solvent protons per catalase molecule. The rate constants *W_ij_* are defined as probabilities of a mutual spin flip per time unit, *e.g.* the contact term *W*_01_ describes the flow of polarization from R0 to R1.

The thermodynamic description of the evolution of nuclear polarization given above has to be extended by structural aspects which are relevant in neutron scattering. The protons within each R*i* are no longer considered as ensembles characterized by a single number *N_i_* but as individuals characterized by their own coordinates and polarization-dependent weight inside the catalase molecule.

We start with the definition of the scattering amplitude of the catalase molecule. In the absence of nuclear polarization, the amplitude *A* ^(0)^(**Q**) of non-interacting particles in dilute solution is given by the difference between the amplitude of the molecule *in vacuo* and the amplitude of the solvent molecules displaced by the volume of the dissolved molecule. **Q** is the momentum transfer in units of ℏ, and *Q* = (4π/λ)sinθ is its modulus, where λ is the neutron wavelength and 2θ is the scattering angle.

In the presence of proton polarization, we proceed in a similar way: the known positions of the protons in the reservoirs associated with the solute constitute the amplitudes *B*_*i*_(**Q**). The protons inside the solvent volume displaced by the solute define *V*(**Q**). The polarization-dependent amplitude is then given by
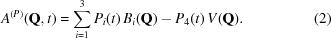
Using the expansion of the scattering amplitude as a series of spherical harmonics, we obtain the intensity of coherent small-angle scattering
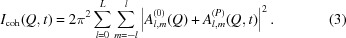
We remember that one may expand an amplitude *A*(**Q**) as a series of spherical harmonics *Y_l,m_*(θ, φ) involving the polar coordinates [*r_n_*, θ_*n*_, φ_*n*_] of the individual atoms (or grid points defining a region) with corresponding scattering lengths *b_n_*:
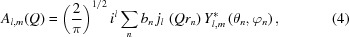
where *j_l_* are spherical Bessel functions of order *l*.

Apart from the intensity of the coherent scattering, experimental neutron scattering data will contain incoherent scattering and background scattering
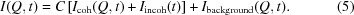
The factor *C* takes into account how the instrument is operated (*e.g.* incident neutron flux, measuring time, detector efficiency) and some properties of the sample, like the concentration of the solute and the transmission of the sample.

The intensity of incoherent neutron scattering is mainly due to the protons (Abragam & Goldman, 1982 ▸; Glätlli & Goldman, 1987 ▸):
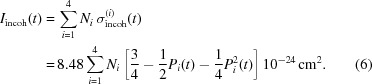
Although deuterons constitute 90% of the hydrogen isotopes of the present sample, their contribution to the intensity of the incoherent scattering is about ten times smaller than that from the protons.

A first estimate of the background scattering intensity can be made by measuring the neutron scattering intensity of the empty refrigerator and the time-dependent transmission of the polarized sample. There may be an additional *Q*- and *t*-dependent scattering intensity, which may be due to a spatially non-uniform proton polarization or to an intrinsic property of the instrument. This is taken into account in two different ways.

(i) The time-dependent background is described by a model like the following: 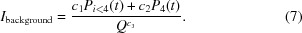



(ii) The time-dependent scattering of a blank sample is subtracted from that of the sample to be investigated. In the absence of a real blank, sample 3 in Table 1 ▸, with a very low concentration of catalase, serves as a substitute.

As the time-dependent part of *I*(*Q*, *t*) is small compared with the total scattering intensity, it is convenient to define its time average
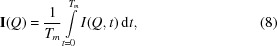
where *T_m_* = 10 s is the length of the cycle. Subtraction of **I**(*Q*) yields the time-dependent function
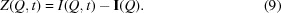
The difference between the calculated *Z*_calc_(*Q*, *t*) and the measured *Z*_exp_(*Q*, *t*) is minimized in an iterative procedure using as variables the scale factor *C*, the rate coefficients *W_ij_*, and – only in case (i) – the coefficients *c*_1_, *c*_2_ and *c*_3_, weighting the intensity of background scattering
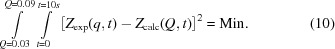
The result of the iteration is stabilized by including two more conditions, concerning the total scattering intensity of the neutron scattering and the variation in the NMR intensity during one cycle, respectively
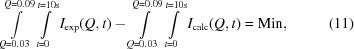


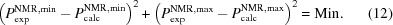
We remember that the proton polarization *P* ‘seen’ by NMR is that of the bulk, *i.e.* that of *P*_3_(*t*) and *P*_4_(*t*). The influence of *P*_1_(*t*) and probably that of *P*_2_(*t*) on the NMR intensity is negligibly small (Leymarie, 2002 ▸).

Equations (10)–(12) [Disp-formula fd12] define the analysis of data from time-resolved polarized neutron scattering from protons in dynamically polarized targets. We replace this somewhat lengthy term by ‘time-resolved proton polarization’ and abbreviate it as TPP.

## 5. Results   

**Figure 4:**
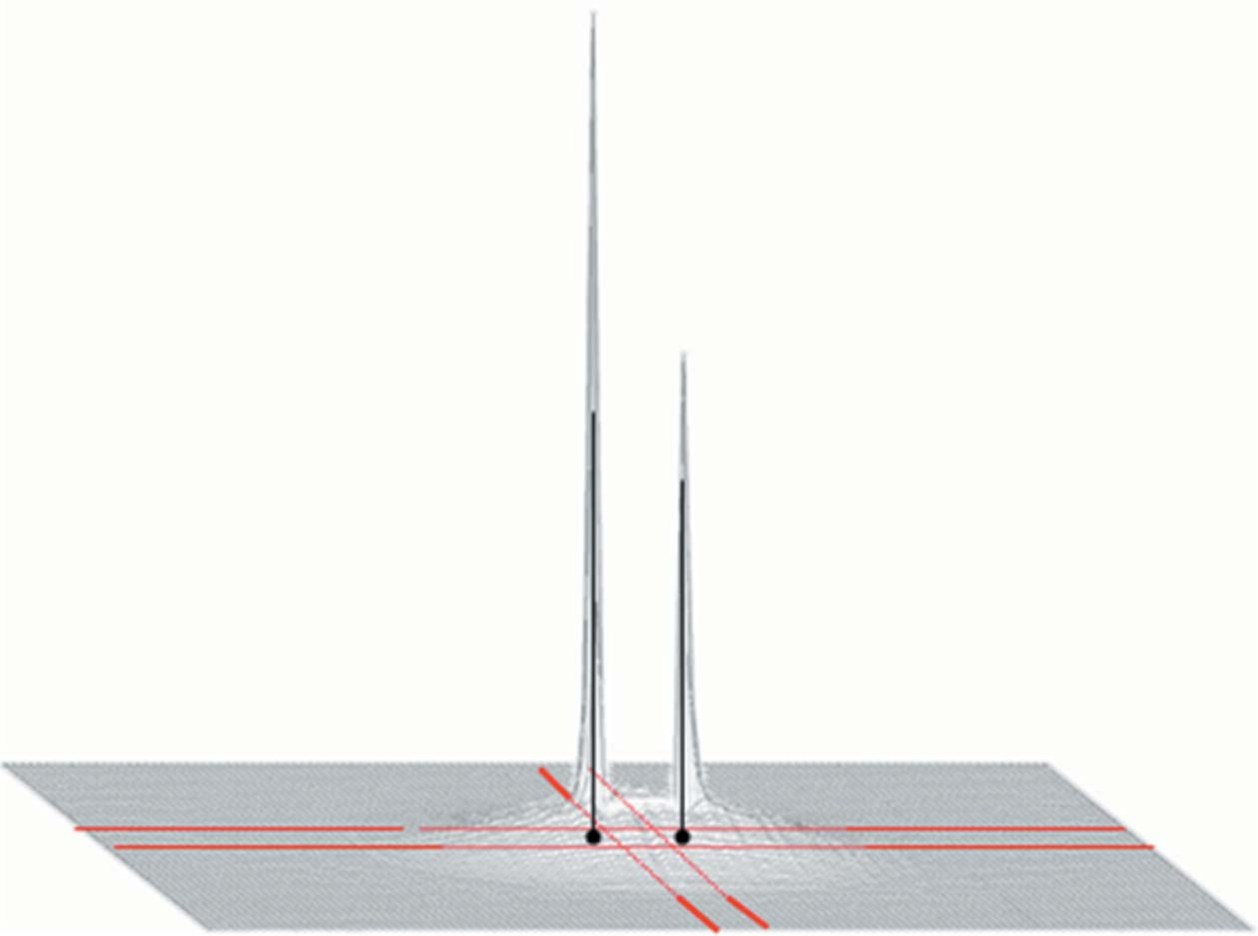
The neutron scattering intensity from catalase as recorded by the area detector of D22. The large peaks on the left and right side of the beamstop are part of the primary beam. It is the neutron beam polarizer about half a metre upstream of the diaphragm at the sample which slightly enlarges the beam in the horizontal direction. The red lines denote the limits of the spherical averaging of the scattering intensity (see Fig. 7).

SANS data from the frozen solutions of catalase in glycerol–water mixtures, as recorded by the area detector of D22, are shown in Fig. 4 ▸. The unexpected large sharp peaks to the left and right of the beam stop are spurs of the primary beam, which has accidentally been enlarged in the horizontal plane by the neutron spin polarizer, less than half a metre upstream of the sample. Initially, it was argued that the intensity of the peaks could serve as an accurate measure of the transmission of the sample. This point will be discussed in more detail below.

**Figure 5:**
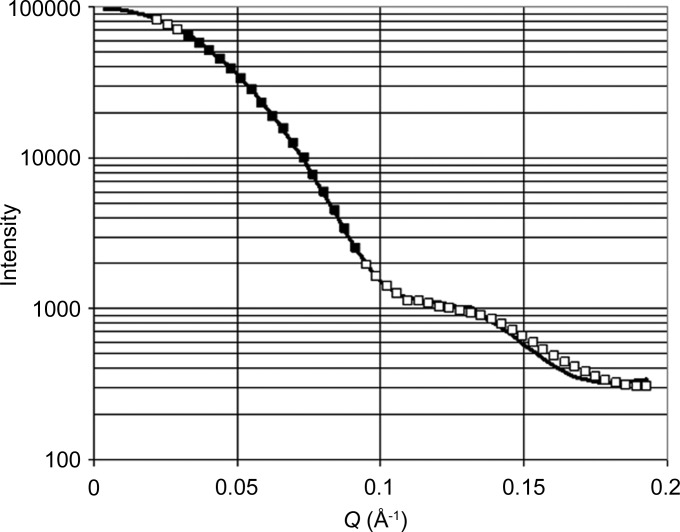
SANS data from a frozen solution of catalase (sample 1). Squares denote measured SANS, and the SANS intensity within the *Q* interval denoted by the filled squares was used for the analysis of time-resolved SANS. The calculated scattering intensity from the catalase model (solid line) agrees with the measured one.

After subtraction of the background (which is approximately the scattering intensity of the empty cryostat) and averaging over radii and time, we obtain **I**(*Q*) shown in Fig. 5 ▸. The scattering curve calculated from the known structure of catalase (Fita & Rossmann, 1985 ▸) agrees with the measured one. Experimental data which lie in the interval 0.03 < *Q* < 0.09 Å^−1^ will be used for the analysis of time-resolved SANS.

The polarization build up for the three frozen solutions of bovine liver catalase (Table 1 ▸) was studied by TPP. Two sequences of DNP and relaxation were used:

(i) 5 s positive DNP followed by 5 s negative DNP, or the inverse sequence.

(ii) 5 s relaxation followed by 5 s negative DNP.

**Figure 6:**
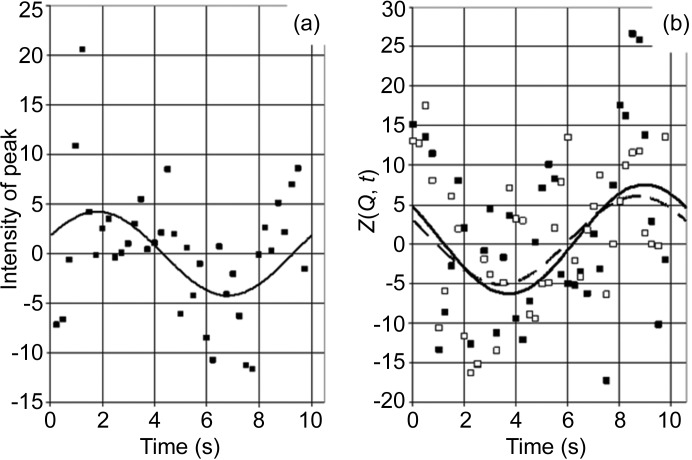
Measured time-dependent neutron scattering intensities. (*a*) Black squares indicate the intensity of the peaks near the beamstop (see Fig. 4 ▸), and the solid line shows the result after a one-dimensional Fourier analysis truncated at |*N*| = 1. (*b*) Black and open squares indicate SANS at *Q* = 0.044 and 0.055 Å^−1^, respectively, and the lines present the time-dependent intensities after a one-dimensional Fourier analysis terminated at |*N*| = 1; the dashed line refers to the open symbols. The relative changes in the intensities in parts (*a*) and (*b*) are 7.0 × 10^−4^ and 4.0 × 10^−4^, respectively. The units of intensity in part (*b*) are as in Fig. 5 ▸.

Let us start with the first case. The intensity of both the prominent peaks near the beam stop (Fig. 6 ▸ *a*) and of the SANS data from catalase (Fig. 6 ▸ *b*) varies during one cycle of DNP in a way which can be described by a sinusoidal function. We try to extract some of the features of these data by a Fourier analysis of their time-dependent part. *Z*_exp_(*Q*, *t*) is then defined as a two-dimensional array of intensity at [*Q*, *m*Δ*t*], with 1 ≤ *m* ≤ *M*, where *M* = 200 is the number of time frames during one cycle of DNP. Omitting Δ*t* = 0.05 s, we have 
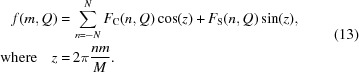

*F*_C_ and *F*_S_ are the Fourier coefficients. A reasonably good fit of the data is achieved by terminating the Fourier series at |*N*| = 1 (Fig. 6 ▸).

As the cycle of DNP starts with polarization in the positive direction, the scattering length density of the catalase mol­ecule will increase, whereas that of the deuterated solvent will hardly change. Hence there is a net decrease in the contrast of the solute with respect to that of the solvent. The intensity of small-angle scattering decreases, as shown in Fig. 6 ▸(*b*). The same is true for the intensity of the incoherent scattering. Hence there will be an initial increase in the transmission of the sample, as shown in Fig. 6 ▸(*a*). The relative change in intensity in the peak area is stronger than that of SANS from catalase.

For hydrogenous substances, the transmission *T* depends largely on the incoherent scattering cross section 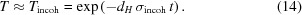
With a proton density of *d*_H_ = 4 ^1^H nm^−3^ (corresponding to a deuteration level of 94%) and a sample thickness *t* = 3 mm, we obtain d*T*/*T* = 0.1 d*P*. With a variation in the polarization of the bulk protons during one cycle of DNP of Δ*P*_NMR_ = (1.0 ± 0.5) × 10^−4^, we expect a variation in the transmission of Δ*T*/*T* = 10^−5^, which is far below the change in the peak intensity of 7 × 10^−4^ (Fig. 6 ▸ *a*). The variation in peak intensity during one cycle of DNP can in no way be related to the proton polarization-dependent transmission of the sample.

The explanation of the time dependence of the peak intensity comes from a Fourier analysis of each pixel of the area detector in terms of *F*_C_(1, *x*, *y*) and *F*_S_(1, *x*, *y*), where *x* and *y* are the coordinates of a pixel. In the peak region the phase angle φ = arctan(*F*_S_/*F*_C_) varies in an intriguingly regular way which points to an intrinsic property of the detector system. As shown in Appendix *B* [App appb] in more detail, the time-dependent response at each pixel varies by 7 × 10^−4^ during one cycle, while the phase angle φ changes from pixel to pixel in a regular way. This finding is interesting for two reasons: first, it shows that this small variation in the response of the detector, probably due to its read-out, is linked to the time frame, and second, that the time-dependent response of the detector system has to be taken into account if one or very few pixels, *e.g.* from crystal diffraction, are considered. It becomes less significant once the integration is performed over larger ensembles, as is the case with radial averaging (see Appendix *B* [App appb]).

**Figure 7:**
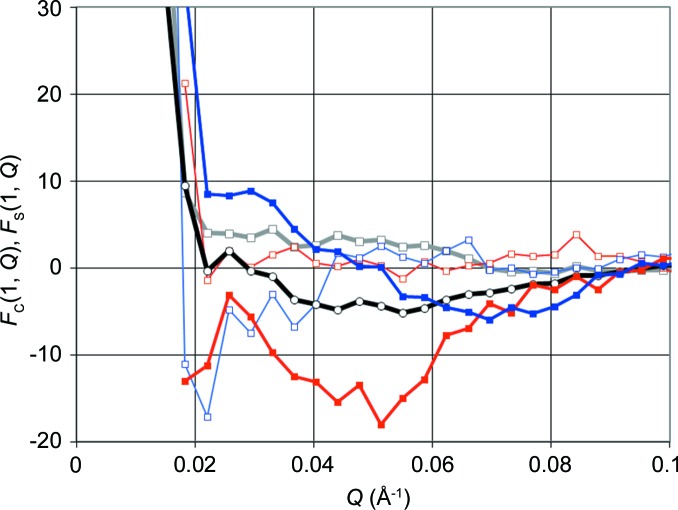
The neutron scattering intensity *Z*_exp_(*Q*, *t*), 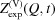 and 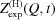 in terms of the Fourier coefficients *F*_C_(1, *Q*) (grey line, open squares), *F*_S_(1, *Q*) (black line, open circles), 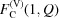 (thin red line, open squares), 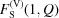 (thick red line, filled squares), 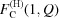 (thin blue line, open squares) and 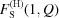 (thick blue line, filled squares). The scale of the Fourier coefficients is the same as in Fig. 5 ▸.

What is the influence of the huge peaks near the beam stop on SANS from catalase? The primary beam not held back by the beam stop gives rise to an increased intensity along the line connecting the peaks (Fig. 7 ▸), which is typical for a two-dimensional cross-wired detector. We therefore restrict the radial averaging on the detector plane to the rows crossing the peaks to obtain 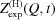. The superscript (H) stands for integration along the horizontal row. The Fourier coefficients 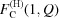 and 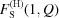 shown in Fig. 7 ▸ are considerably different from *F*_C_(1, *Q*) and *F*_S_(1, *Q*) obtained from the conventional *Z*_exp_(*Q*, *t*). Apparently, the peaks are leaving their mark on the intensity of time-resolved SANS. This is less pronounced with 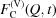 and 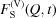 obtained by integration along vertical columns lying between the two peaks (Fig. 4 ▸).

**Figure 8:**
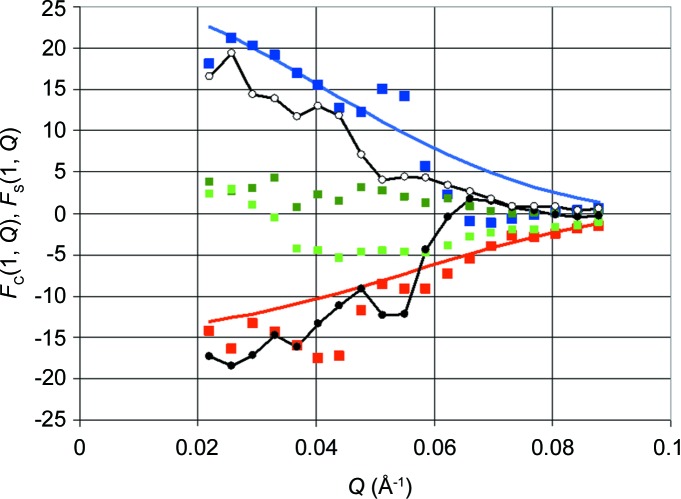
Fourier coefficients *F*_C_(1, *Q* and *F*_S_(1, *Q*) of *Z*_exp_(*Q*, *t*) and *Z*_calc_(*Q*, *t*). For sample 3 (the so-called blank), the black line with filled circles denotes 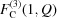 and the black line with open circles denotes 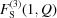. For sample 1, the dark-green filled squares denote 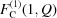 and the light-green filled squares denote 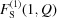. The difference between sample 1 and sample 3 is indicated by blue filled dots for 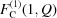 − 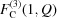 and by red filled squares for 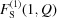 − 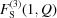. For the models, the solid blue line indicates *F*_C_(1, *Q*) and the solid red line indicates *F*_S_(1, *Q*).

Assuming a constant performance of the detector system, the background scattering intensity will be observed with each sample of catalase. It can be seen most clearly with a sample having a very low concentration of catalase, like sample 3 in Table 1 ▸. For *Q* < 0.05 Å^−1^, 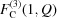 and 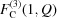 of sample 3, shown in Fig. 8 ▸, are similar to 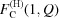 and 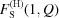 in Fig. 7 ▸. Sample 3 will thus serve as a blank sample, as suggested above.

The analysis of *Z*_exp_(*Q*, *t*) as described in Section 4 uses

(i) the known structure of catalase;

(ii) the preliminary assumption of tyr-369 as a radical;

(iii) the size of the reservoirs R1 and R2 with radii fixed to 5 and 10 Å, respectively;

(iv) the time-dependent intensity of the background as described above.

The variables used to achieve an agreement of the time-dependent neutron scattering intensity calculated from the model, *Z*_calc_(*Q*, *t*), with the experimental data, *Z*_exp_(*Q*, *t*), are

(i) the transition probabilities *W_ij_* defined in equation (1)

(ii) the coefficients *c*_1_, *c*_2_ and *c*_3_ as defined by equation (7) for the case of background only (Section 4).

Each of the four subunits of BLC contains 20 tyrosines in its chain of 506 amino acids (Fita & Rossmann, 1985 ▸). From EPR it is known that one tyrosine has become a tyrosyl radical (Ivancich *et al.*, 1997 ▸). A detailed analysis of the EPR spectrum suggests that the amino acid with the running number 369, tyr-369, has been converted into a tyrosyl (Svistunenko & Cooper, 2004 ▸). As a first step we will take this result for granted and use it to compare the calculated *Z*_calc_ (*Q*, *t*) with the experimental *Z*_exp_(*Q*, *t*). Then, in a second step, the other 19 tyrosines will be subjected to the same procedure. A comparison of the r.m.s. deviations will lead to the most probable site(s) of the unpaired electron.

The conversion of tyr-369 into a radical state is incomplete. For sample 1 it amounts to 0.58 (Table 1 ▸). Each catalase molecule will have between zero and four tyrosyl radicals. The number of tyrosyls per molecule is then described by a bi­nomial distribution (Table 2 ▸). As both *W*_01_ and *W*_12_ depend on the inter-radical distance (see Appendix *A* [App appa]), each configuration of tyrosyls inside a catalase molecule should be treated separately with its proper *W*_*ij*_. To keep the computing time within acceptable limits, we consider two sets of configurations: (i) molecules with more than one radical, and (ii) molecules with no more than one radical. As shown in Appendix *A* [App appa], catalase molecules with a single radical will hardly contribute to DNP. Thus, catalase molecules of class (ii) are considered to be inactive (Table 2 ▸).

Now let us turn to catalase molecules with more than one radical. Taking sample 1 with 58% of its tyrosine-369 converted to a radical state, an occupancy of 67% is obtained for the ‘active’ catalase molecules. The number of protons in R1 and R2 used for the solution of the differential equations (1) is adapted to this occupancy, *i.e.* the number of protons *N*_1_ = 92 is reduced to *N*_1_(used) = 62 (Table 3 ▸).

As the experimental data are very noisy, we prefer to start from *Z*_exp_(*Q*, *t*) presented as Fourier series, either in the form of a one-dimensional Fourier series given by equation (13), or as a two-dimensional Fourier series. The following part of the analysis starts from a presentation of the neutron scattering data as a two-dimensional Fourier series. *Z*_exp_(*Q*, *t*) is then defined as a two-dimensional array of intensities at [*m*_1_Δ*Q*, *m*_2_ *Δt*] with 1 ≤ *m*_1_ ≤ *M*_1_ and 1 ≤ *m*_2_ ≤ *M*_2_. Omitting Δ*Q* = 0.0037 Å^−1^ and Δ*t* = 0.05 s, we have 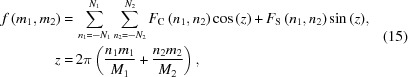
where *M*_1_ = 16 (the number of full squares in Fig. 4 ▸) and *M*_2_ = 200 (the number of time frames per cycle of DNP). *N*_1_ and *N*_2_ should not be confused with the number of hydrogen atoms per reservoir, *N*_*i*_.

**Figure 9:**
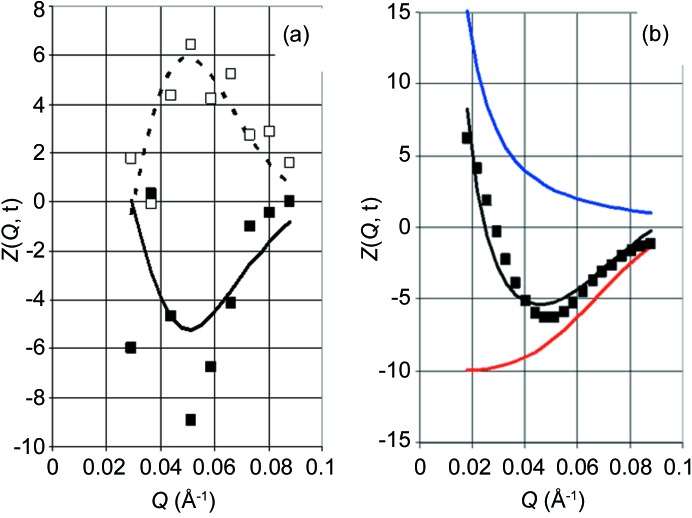
Time-resolved neutron scattering *Z*_exp_(*Q*, *t*) for sample 1 *versus Q*. (*a*) At *t* = 2.5 s and 7.5 s in the lower and upper part of the figure, respectively. Symbols represent experimental data averaged over −1 < *t* < 1 s and −0.01 < *Q* < 0.01 Å^−1^, and the lines are the experimental data after two-dimensional Fourier analysis. (*b*) The filled black squares indicate *Z*_exp_(*Q*, *t*) after two-dimensional Fourier analysis at *t* = 2.5 s and the solid black line denotes *Z*_calc_(*Q*, *t*) at *t* = 2.5 s. Also shown is the decomposition of *Z*_calc_(*Q*, *t*) into SANS (solid red line) and background (solid blue line). The units of the intensity are as in Fig. 5 ▸.

In view of the poor statistics of the experimental data, only a few Fourier coefficients *F*_C_ and *F*_S_ are used. The Fourier series describing the time dependence of *Z*_exp_(*Q*, *t*) is terminated at |*N*_2_| = 1, as was done for the one-dimensional Fourier series of *Z*_exp_(*Q*, *t*). For a description of the *Q*-dependence of *Z*_exp_(*Q*, *t*), more Fourier coefficients, up to |*N*_1_| = 3, are used. Note that, in this case, the series is based on *Z*_exp_(*Q*, *t*) and *Z*_exp_(−*Q*, *t*). A result of the two-dimensional Fourier analysis of *Z*_exp_(*Q*, *t*) from the more concentrated catalase sample (sample 1 in Table 1 ▸) is shown in Fig. 9 ▸(*a*). At *Q* = 0.05 Å^−1^, there is a strong variation in the intensities between 2.5 s and 7.5 s, which points to the presence of a sine wave. The same feature is present in the one-dimensional Fourier series of *Z*_exp_(*Q*, *t*) shown in Fig. 7 ▸, where *F*_S_(1, *Q*) and 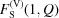 have their extremes at the same *Q* values.

**Figure 10:**
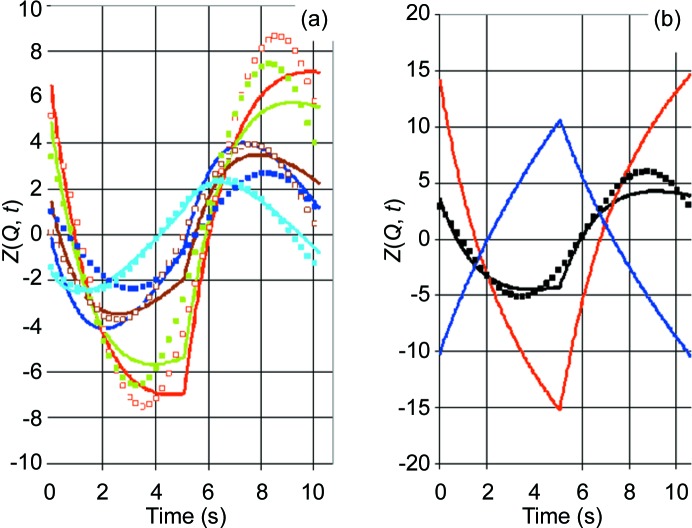
(*a*) Intensity of neutron scattering *Z*(*Q*, *t*) *versus* time at various *Q* (in Å^−1^): dark blue 0.033, red 0.044, green 0.055, brown 0.066 and light blue 0.077. Symbols represent experimental data after two-dimensional Fourier analysis, and lines are *Z*_calc_(*Q*, *t*) calculated from the model. (*b*) The decomposition of *Z*_calc_(*Q*, *t*) (solid black line) into SANS (solid red line) and background (solid blue line) at *Q* = 0.05 Å^−1^; the solid black squares represent *Z*_exp_(*Q*, *t*) after two-dimensional Fourier analysis. Units are as in Fig. 5 ▸.

At *Q* = 0.03 Å^−1^, the variation in intensity due to proton polarization is neutralized by the time-dependent response of the detector system (Fig. 6 ▸ *a*). Using the background defined by equation (7), *Z*_exp_(*Q*, *t*) in terms of the two-dimensional Fourier series is reasonably well approximated by *Z*_calc_(*Q*, *t*) derived from the model defined in Section 4 (Fig. 9 ▸ *b*). The same figure shows the decomposition of *Z*_calc_(*Q*, *t*) into the coherent scattering intensity from catalase and the background. There is a strong increase in the background at smaller *Q* which looks similar to that of 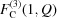 and 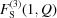 shown in Fig. 8 ▸. The variation in *Z*(*Q*, *t*) with time for various *Q* is shown in Fig. 10 ▸. At *Q* = 0.05 Å^−1^, the contributions of SANS and background to *Z*_calc_(*Q*, *t*) are comparable and opposite in sign (Fig. 10 ▸ *b*).

**Figure 11:**
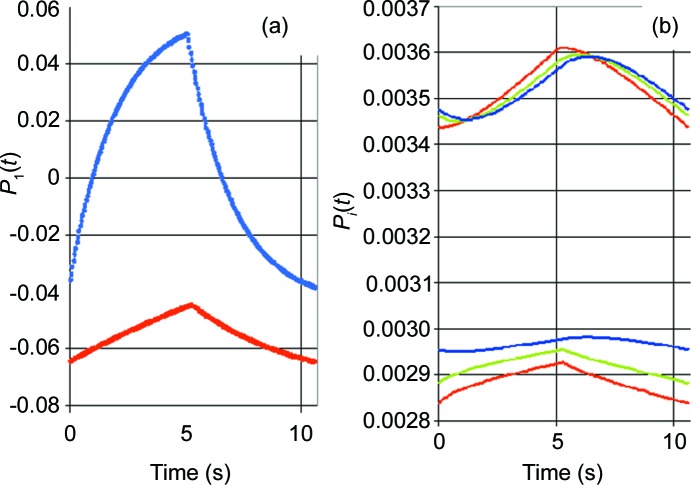
Proton polarization *versus* time during one cycle of DNP. (*a*) *P*_1_(*t*) in R1, with the blue line indicating the sequence DNP positive followed by DNP negative and the red line indicating the sequence 5 s relaxation followed by DNP negative. (*b*) Red lines indicate *P*_2_(*t*), green lines *P*_3_(*t*) and blue lines *P*_4_(*t*); in the upper part of the diagram the sequence is DNP positive then DNP negative, while that in the lower part is relaxation followed by DNP negative.

We return to the proton polarization during one cycle of DNP, shown in Fig. 11 ▸. While the close protons in R1 respond immediately to a change in the direction of DNP, those in more remote reservoirs react more slowly. At the onset of positive DNP, the polarization of the solvent protons continues to decrease for another 1.5 s, before slowly changing its direction to more positive polarization. While the polarization of the close protons of R1 varies between −3.7% and 4.8%, the polarization of the protons in the remote R*i*, and even in the neighbouring R2, varies only slightly at values close to the thermal equilibrium proton polarization of *P*_e_ = 0.35% valid for *T* = 1 K and *B* = 3.5 T. Nevertheless, their contribution to the time-dependent total polarization can be considerable because of the huge number of protons, particularly in R3 and R4 (Table 3 ▸). The variation in the total proton polarization of Δ*P* = (0.01 ± 0.005)% obtained from time-resolved neutron scattering appears to agree with that obtained from the simultaneous measurement of time-resolved proton NMR.

The change in polarization of 62 protons [*N*_1_(used) in Table 3 ▸] by 8.5% corresponds to 5.3 polarized close protons per catalase molecule. After correction for the occupancy of 0.67 typical for the set of active configurations shown in Table 2 ▸, the number of polarized protons increases to 7.9. During one cycle of DNP, the number of polarized protons per tyrosyl will change by *N*_p_ = 2.0 ± 0.5. The corresponding number of polarized protons in R2 is about 0.1.

Now we turn to the second case, *i.e.* 5 s relaxation followed by 5 s negative DNP. The evolution of the proton polarization with time is shown in Fig. 11 ▸. Contrary to the previous example, there are only two counteracting forces: the drift of proton polarization towards *P*_e_ = 0.35% at thermal equilibrium, and the subsequent negative proton polarization by DNP. On the whole, the variations in proton polarization are smaller than in the previous case. The variation of *P*_1_(*t*) between −6.4% and −4.5% reflects the variation in the number of polarized protons by 1.2 per catalase molecule which, after corrections, corresponds to 0.4 polarized protons per tyrosyl. As for the bulk protons, their polarization remains positive and only slightly below *P*_e_. This result is in agreement with an earlier observation shown in Fig. 2 ▸.

The evolution of the close proton polarization with time in R1 shown in Fig. 11 ▸ can be approximated by an exponential function of time characterized by a time constant τ. For alternating directions of DNP we obtain τ = (1.5 ± 0.5) s, whereas longer characteristic times are typical for a sequence of relaxation and negative DNP.

So far, the two-dimensional Fourier series of *Z*_exp_(*Q*, *t*) has been compared with *Z*_calc_(*Q*, *t*). In the following, *Z*_exp_(*Q*, *t*) will be described by a one-dimensional Fourier series. Furthermore, we use sample 3 as a blank sample, *i.e.* *Z*_exp_(*Q*, *t*) = 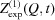 − 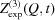. There is no need for a background defined by equation (7). In terms of Fourier coefficients we have
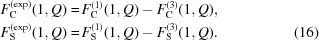
Note that 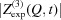 is larger than 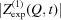 at small *Q*, which confirms the relatively large background seen in Figs. 9 ▸(*b*) and 10 ▸(*b*), where equation (7) has been used.

The analysis is based on equations (10)–(12) [Disp-formula fd12] as before. For an easier appraisal of the result, *Z*_exp_(*Q*, *t*) in terms of 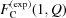 and 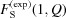 is compared with *Z*_calc_(*Q*, *t*) in terms of 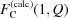 and 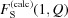 in Fig. 8 ▸. The results differ slightly from those based on a two-dimensional Fourier series. The main differences are that:

(i) The number of close protons polarized in excess, *N*_P_, per tyrosyl is distributed among R1 and R2 in a different way: 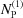 = 1.6 in R1, and 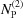 = 0.6 in R2.

ii) The characteristic time τ for the build-up of the local proton polarizations of 1.0 s falls slightly below the 1.5 s mentioned above.

The total number of close protons in R1 and R2 is close to that obtained from *Z*_exp_(*Q*, *t*) in terms of a two-dimensional Fourier series.

**Figure 12:**
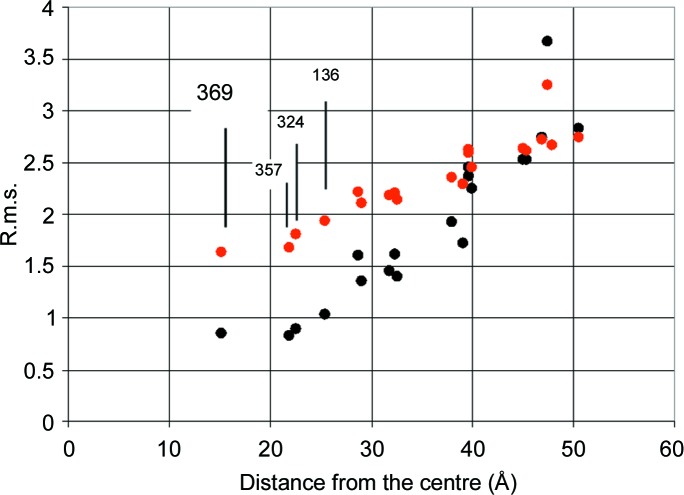
The r.m.s. deviation of *Z*_calc_(*Q*, *t*) of the model from that of the measured *Z*_exp_(*Q*, *t*) for the 20 tentative tyrosyl sites. Black filled circles are data from the two-dimensional Fourier analysis and red filled circles are data from the one-dimensional Fourier analysis. The lowest r.m.s. values are obtained with tyr-369 together with tyr-357 and tyr-324 as radical sites.

**Figure 13:**
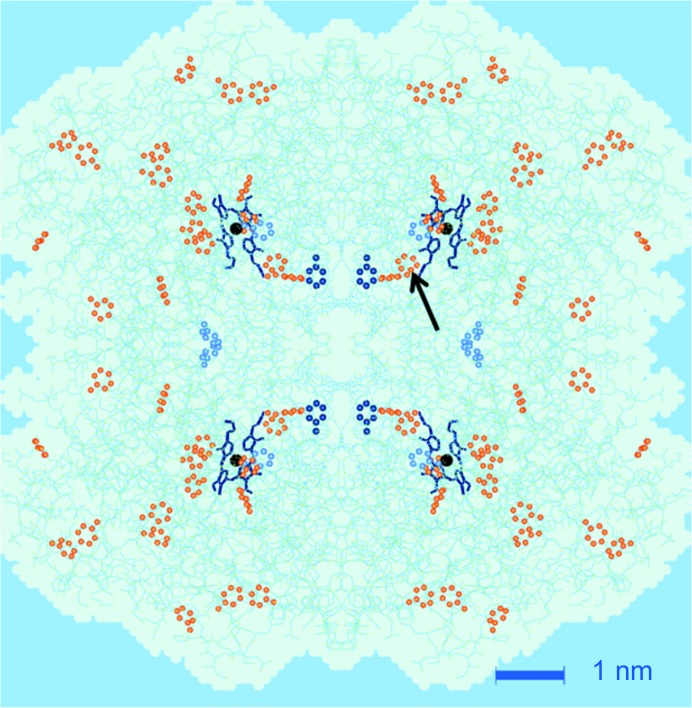
The tetramer bovine liver catalase molecule, with its tyrosines presented by their phenyl rings, most of them coloured orange. From neutron scattering it appears that one of the following tyrosines (coloured blue) may have become a radical: tyr-324, tyr-357 or tyr-369. Tyr-136 (marked by an arrow) is another slightly less probable candidate. From EPR (Svistunenko & Cooper, 2004 ▸), tyrosyl-369 is suggested (dark blue, closest to the centre of the catalase molecule). Tyr-357 is very close to the iron atom (black filled circles) of the porphyrin ring.

A final point concerns the detection of the tyrosine converted into a radical. So far, our analysis of time-resolved neutron scattering started from tyr-369 as the most likely radical site. What is the answer from neutron scattering? The answer comes from a test subjecting all 19 remaining tyrosines as possible radical sites to the same analysis as for tyr-369. The minimum r.m.s. values defined by equation (10) increase with the distance of the hypothetical radical site from the centre of the catalase molecule (Fig. 12 ▸). This is true for both sets of input data, from one- and two-dimensional Fourier analysis, respectively. Tyr-369 together with tyr-357 and tyr-342 are equally good candidates for a radical site. Tyr-136, with a slightly higher r.m.s. deviation, might be included as well. TPP selects four tyrosines out of 20 per subunit as possible radical sites (Fig. 13 ▸).

## 6. Discussion   

A feature common to both sequences of DNP presented above is the clear difference between the protons in R1 and those of the other reservoirs. The protons in reservoir R2, which were tentatively introduced as a mixture of close and bulk protons, appear to belong only to the class of bulk protons, in agreement with Fig. 11 ▸. However, the results obtained from *Z*_exp_(*Q*, *t*) in terms of a one-dimensional Fourier analysis suggest a larger change in the proton polarization in R2 during one cycle of DNP, putting these protons on the side of the close protons. The polarization of the close protons is maintained by a magnetic nuclear spin diffusion barrier somewhere inside R2 that is at a distance of between 5 and 10 Å from the unpaired electron of the radical.

There is a variation of 2 ± 0.5 polarized protons close to a tyrosyl radical during one cycle of alternating direction of DNP. This is about half the number of polarized protons in R1 observed with a solution of EHBA–Cr^V^ in a glycerol–water mixture (see Appendix *A* [App appa]). As EHBA–Cr^V^ is known for its very efficient support of DNP, the outcome from this first attempt with a tyrosyl radical in catalase is promising. For structural studies, it is of interest to compare the change in the scattering length of R1 due to the polarization of its protons with those of competing techniques like magnetic neutron scattering. The polarization of two protons corresponds to a change in scattering length of 2.9 × 10^−12^ cm, which is about ten times larger than the magnetic scattering length of an unpaired electron. It is also well above the average nuclear scattering length of 0.6 × 10^−12^ cm for non-hydrogen atoms present in organic matter.

**Figure 14:**
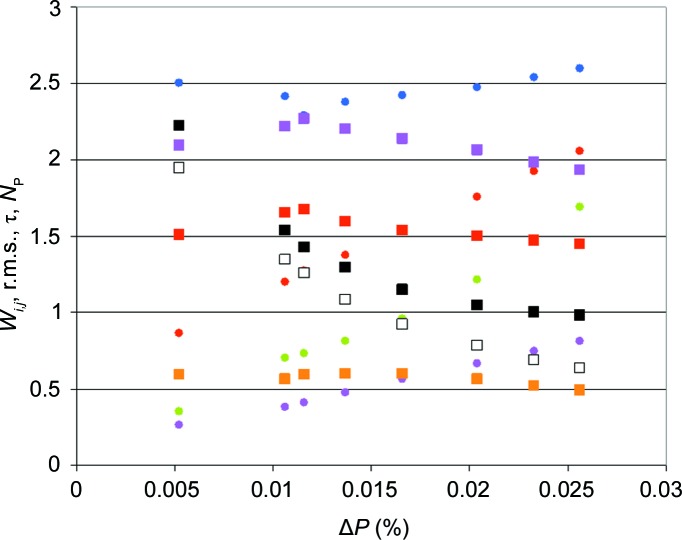
The transition probabilities *W_ij_*, r.m.s., τ and the number *N*_P_ of polarized protons per tyrosyl *versus* Δ*P* from NMR at minimum r.m.s., defined by equations (10)–(12) [Disp-formula fd12]. Black squares indicate the r.m.s., white squares the characteristic time τ (in seconds), red, yellow and pink squares the number of protons polarized in excess in R1, R2 and R1+R2, respectively, and red, pink, green and blue circles the transition probabilities *W*_01_, *W*_12_/100, *W*_23_/1000 and *W*_34_/10000, respectively.

The sources of error in the number of polarized protons quoted above are both statistical and systematic. We restrict the discussion to the error in the measurement of the faint NMR intensity from the protons of the catalase sample and its repercussions on the analysis of time-resolved neutron scattering data. We remember that the proton polarization is close to its value at thermal equilibrium, *P*_e_ = 0.35%, and that the periodic change in the polarization of the bulk protons measured by NMR amounts to Δ*P* = (0.010 ± 0.005)%. In order to test the influence of the error in the NMR measurement, we perform the least-squares procedure defined by equation (10) for a number of different values of Δ*P*, starting from the one-dimensional Fourier series of *Z*_exp_(*Q*, *t*). The number of polarized protons, the transition probabilities, the characteristic time τ and the r.m.s. deviation change with Δ*P* to different degrees (Fig. 14 ▸). A good fit of the neutron scattering data would be obtained with Δ*P* = 0.02%, which exceeds the measured Δ*P* = 0.01%. As a compromise between data from NMR and neutron scattering, we fix Δ*P* to 0.013%. Then we obtain 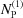 = 1.6 in R1 and 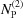 = 0.6 in R2, as cited above. While the choice of Δ*P* has a relatively small influence on *N*_P_, the characteristic time τ varies strongly with Δ*P*.

As for the determination of the site of a radical in a protein from time-resolved proton polarization (TPP) observed by SANS, a rigorous treatment will start from subdividing the volume of the catalase molecule into cubes of 1 nm^3^, for instance. Each of the 75 cubes needed to fill one subunit of catalase would be subjected to the same procedure which has been applied to tyr-369. The result would be a radical density map in the strict sense. We reduced the number of items to be tested by choosing the 20 tyrosines as the only possible radical sites, at the expense of structural resolution. As the tyrosines are fairly evenly distributed all over the catalase molecule, the result of this strategy may still be called a radical density map. How precise is it? Using data at *Q* < 0.1 Å^−1^, the method of TPP using SANS provides the radial distribution of the radial density map. An angular resolution in terms of the polar angles θ and φ seems to be out of reach, at present.

**Figure 15:**
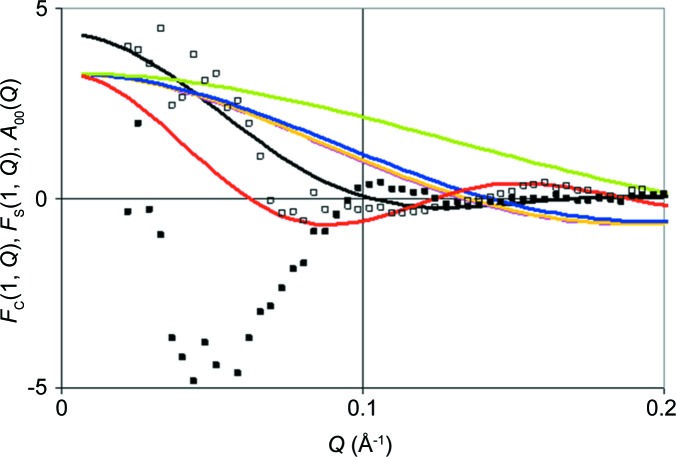
The Fourier coefficients *F*_C_(1, *Q*) and *F*_S_(1, *Q*) of *Z*_exp_(*Q*, *t*) compared with the amplitudes 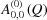 and 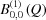 of various tyrosines. White squares indicate *F*_C_(1, *Q*), black squares *F*_S_(1, *Q*) and the black line 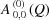 of catalase, while the red, pink, yellow, blue and green lines indicate 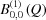 of tyrosines tyr-446, tyr-136, tyr-324, tyr-357 and tyr-369, respectively.

A comparison of the *Q*-dependent Fourier coefficients *F*_C_(1, *Q*) and *F*_S_(1, *Q*) with the radial (or monopole) functions *A*_00_(*Q*, *t*) of catalase and some of its tyrosines at *Q* > 0.2 Å^−1^, shown in Fig. 15 ▸, sheds some light on the limits of structural resolution from TPP using SANS. Let us focus on their zeroes. *Z*_exp_(*Q*, *t*) will be zero if both *F*_C_(1, *Q*) and *F*_S_(1, *Q*) are zero. *Z*_calc_(*Q*, *t*) will be zero if products of the type 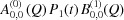 are zero. Here, we have assumed that 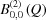 ≃ 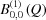 and 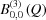 ≃ 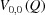 ≃ 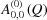. At *Q* < 0.1 Å^−1^ there is no zero of *Z*_exp_(*Q*, *t*), which can be attributed to one of the monopoles, 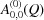 or 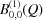, obtained from the model. This means that possible radical sites are not more than 30 Å away from the molecular centre, which is compatible with the model shown in Fig. 13 ▸. The zeroes of 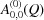 co­incide with those of *Z*_exp_(*Q*, *t*) at *Q* = 0.1 Å^−1^ and *Q* = 0.17 Å^−1^. Some of the zeroes of the monopole functions 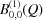 of tyrosines close to the centre are well separated and could be distinguished with more accurate experimental data. Moreover, at *Q* > 0.1 Å^−1^ the scattering intensity from higher multipoles becomes significant. Their presence could help to find the angular coordinates θ and φ of radical sites as well.

## 7. A crystallographic outlook   

The determination of a radical density map of proteins can be achieved in a straightforward way by TPP using protein crystallography. The application of the difference Fourier method to time-resolved neutron diffraction data allows us to follow the evolution of the radical density map in space and time.

Unlike TPP using SANS, which is a model-based technique, TPP with a radical-doped crystal does not require the introduction of the reservoirs R1–R4 beforehand. Ideally, the sequence of difference Fourier maps obtained for different times of the cycle of DNP is sufficient to show the proton polarization emerging at the radical sites and its propagation throughout the unit cell of the crystal. In a second step, this sequence of radical density maps may be analysed in terms of reservoirs which communicate with each other *via* the transition probabilities *W_ij_* described above. This step may have the additional merit of a refinement of the time-dependent radical density map.

In the absence of data from TPP from a crystal, we will give an estimate of the outcome of such an experiment on the basis of the data reported above. The mathematical description of TPP in crystallography is essentially that given in Section 4, except for the amplitude of the coherent scattering, where the double sum and all that goes with it have to be replaced by exp(*i* **Qr**_*n*_). The time-dependent intensity of the diffracted beam in terms of the indices [*h*,*k*,*l*] of the diffraction peaks is then obtained as 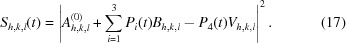
Let us assume that crystals of a material like bovine catalase with some of its tyrosines in a radical state would be at hand, and that we would have access to a facility allowing TPP at a powerful neutron source for a reasonably long time. We will try to make an estimate of the outcome of such an experiment on the basis of the results from TPP with bovine catalase reported above.

The time dependence of the intensity of the diffraction peaks will follow a combination of *P_i_*(*t*) shown in Fig. 11 ▸. While at low resolution the diffracted intensities show an uninterrupted increase or decrease during each half cycle of DNP, the time dependence of high-resolution reflections may look quite different.

**Figure 16:**
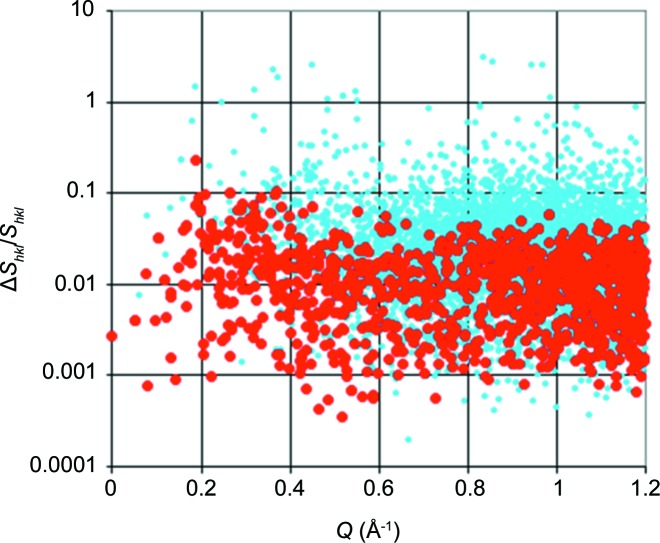
The relative variation in the diffracted intensity Δ*S*_*hkl*_/*S_hkl_* for Bragg reflections up to 5 Å resolution during one cycle of DNP *versus* *Q*. Data points in red are *S_hkl_* above the average. The *S_hkl_* data were derived from the crystal structure of bovine catalase (Fita & Rossmann, 1985 ▸).

In order to give an estimate of the feasibility of such an experiment, we present a survey over the variation in intensity of the Bragg reflections during one cycle of DNP up to a structural resolution of 5 Å. Fig. 16 ▸ shows the relative change in the diffracted intensity Δ*S*_*hkl*_(*t*) during one cycle of DNP divided by the time-averaged intensity *S_hkl_*. As reflections with *S_hkl_* close to zero might give rise to exceptionally high relative intensities, even at modest Δ*S*_*hkl*_, we exclude them by presenting only those with *S_hkl_* above the average (in red in Fig. 16 ▸). A number of them show a relative change in intensity close to 10%. We mention them by their indices *h*, *k*, *l*: [550], [150], [023], [700], [113], [450] *etc*. The time-dependence of these reflections will be useful for the optimization of the TPP method.

## 8. Conclusions   

Time-resolved polarized neutron scattering from dynamically polarized protons (TPP) using SANS is able to image radicals in large structures, like proteins or membranes. Applied within the framework of SANS, the structural resolution will be limited to the diameter of the domain of close protons, *i.e.* to approximately 10 Å. Attempts should be made to extend the method to TPP-based neutron crystallography. This would not only locate the radical sites with a presumably much higher sensitivity, but also focus on the spatial distribution of the protons close to an unpaired electron.

## Figures and Tables

**Figure 17:**
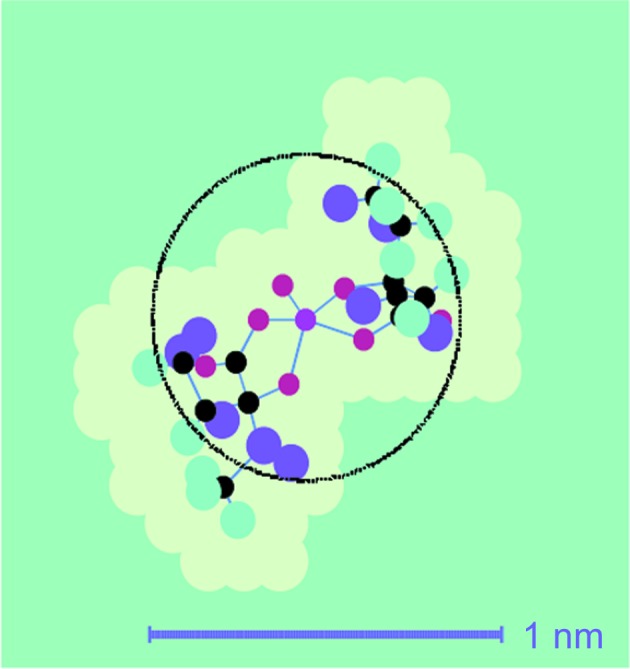
The chromium(V) complex anion, (2-hydroxy-2-ethylbutyrato) oxo­chromate(V), abbreviated as EHBA–Cr^V^, C_12_O_7_H_20_Cr. The central Cr atom is surrounded by five O atoms (red). C atoms are shown as small black spheres. Large spheres represent H atoms. The close H atoms of the solute within a spherical R1 (dotted circle) are shown in violet.

**Figure 18:**
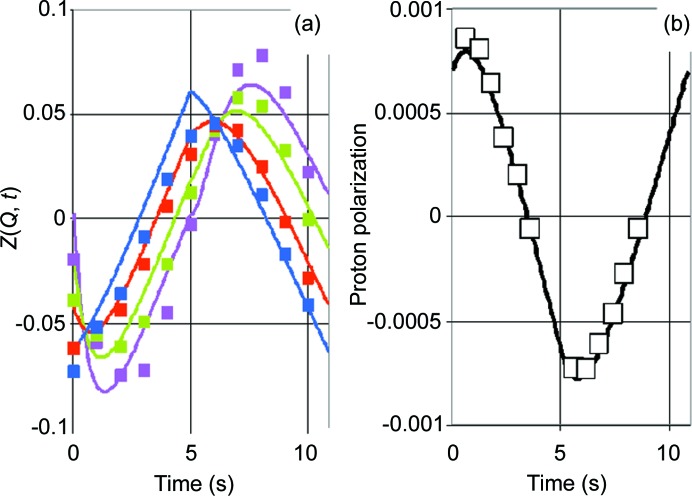
Time-resolved neutron scattering *Z*(*Q*, *t*) at various *Q*, and the polarization of bulk protons *versus* time from EHBA–Cr^V^ (*c* = 1.53 × 10^19^ ml^−1^) in a mixture of glycerol/water. (*a*) *Z*_exp_(*Q*, *t*) is presented as two-dimensional Fourier series terminated at *N*_1_ = 1 and *N*_2_ = 1: pink 0.30, green 0.45, red 0.58 and blue 0.70, with the respective lines denoting *Z*_calc_(*Q*, *t*). An average value of 620 a.u. has been subtracted from *I*(*Q*, *t*) to obtain *Z*(*Q*, *t*). (*b*) Open squares and the black line denote the bulk proton polarization from NMR and from *Z*_calc_(*Q*, *t*), respectively.

**Figure 19:**
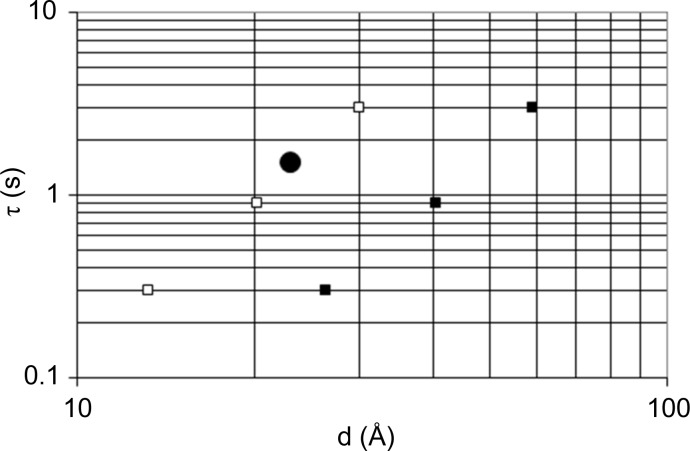
Characteristic time τ *versus* inter-radical distance. Black circle: tyrosyl of catalase; solid black squares: EHBA–Cr^V^; open squares: EHBA–Cr^V^ molecules at their effective distance *D*_e_, which is smaller than the intermolecular distance *D* by a factor of approximately 2. This factor is obtained from 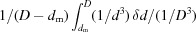, where *d*_m_ is the mol­ecular diameter and *D* is the average distance between neighbouring molecules. For the calculation of *D*, the EHBA–Cr^V^ molecules were placed on grid points of a cubic lattice.

**Figure 20:**
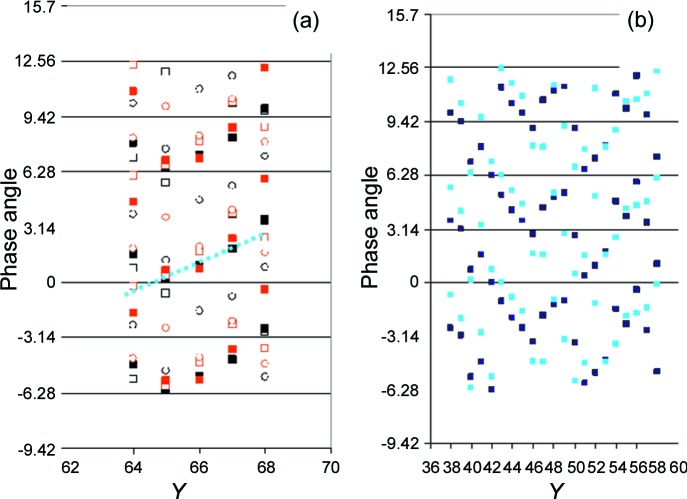
The time-dependent response of the area detector *versus* column *Y*. (*a*) Pixels close to the beam stop (Fig. 4 ▸), with *X* coordinates as follows: left of the beam stop open squares 59, black filled squares 60, open circles 61, right of the beam stop red open squares 70, red filled squares 71, red open circles 72. At *X* = 60 or 71 and 65 ≤ *Y* ≤ 67, the intensity of the peak largely exceeds that of SANS from catalase. In this region, the phase angle (see text) for a given column number *X* increases by 0.95 (= 54°) on stepping from one row to the next, as indicated by the dotted line. (*b*) The intensity of the incoherent neutron scattering from EHBA–Cr^V^ in a protiated solvent (see Appendix *A* [App appa]). There is little variation in the intensity with time and scattering angle. It is for this reason that a regular change in the phase angle (see text) can be observed over several adjacent *Y* channels everywhere on the area detector. Filled black squares: *X* = 40; filled blue squares: *X* = 41. As the systematic change in the phase angle may extend over more than 2π, each phase angle is shown three times shifted by multiples of 2π.

**Table 1:** Samples of bovine liver catalase The occupancy of tyrosyl as used for the analysis of the neutron scattering data is the ratio tyrosyl:haem determined by EPR.

Name	Concentration (g l^−1^)	Haem (m*M*)	Tyrosyl (m*M*)	Tyrosyl:haem ratio
Sample 1	31.5	0.515	0.300	0.583
Sample 2	15.5	0.258	0.200	0.775
Sample 3	7.6	0.129	0.065	0.504

**Table 2:** The repartition of the tyrosyl radicals (black filled circles) on catalase molecules of sample 1 With an occupancy *p* = 0.58, the probability of *k* tyrosyls on a catalase molecule obeys the binomial distribution *p*_4_(*k*) = 
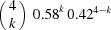
 shown in the last column.

Number of tyrosyls per catalase molecule and their activity	Arrangement of the tyrosyls on the four subunits of catalase	Binomial distribution *p*_4_(*k*), *k* = number of tyrosyls
0, inactive	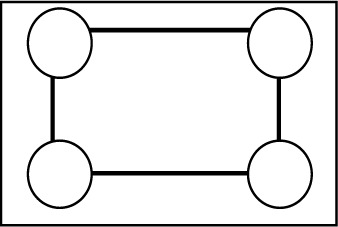	0.031
1, inactive	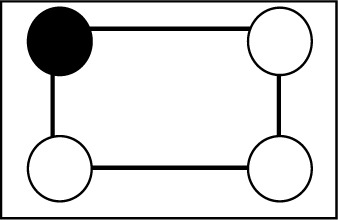	0.172
2, active	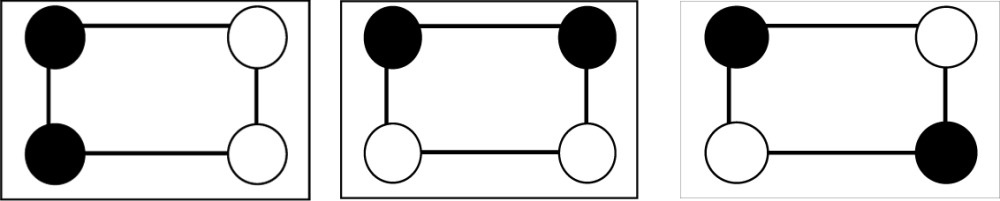	0.356
3, active	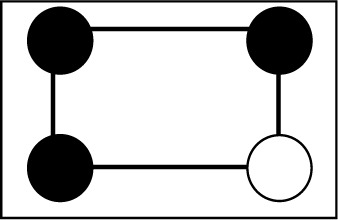	0.328
4, active	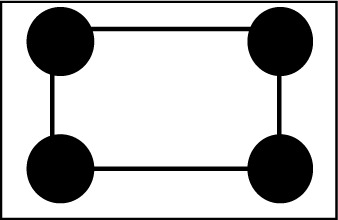	0.113

**Table 3:** The reservoirs R*i* of sample 1 and the transition probabilities governing the cycle of positive and negative DNP *N*_*i*_ is the number of protons in reservoir R*i* of the catalase molecule. *N*_*i*_(used) is the number of protons close to active tyrosyls defined in Table 2 ▸. *W_ij_* are the transition probabilities of proton polarization between the reservoirs R*i* and R*j*.

Reservoir	*N*_*i*_	*N*_*i*_ (used)	*W_ij_* (s^−1^)
R1, 0 ≤ *r* ≤ 5 Å	92	62	*W*_01_ = 1.5
R2, 5 ≤ *r* ≤ 10 Å	612	425	*W*_12_ = 50
R3, catalase	11700	11700	*W*_23_ = 1000
R4, solvent	18000	18000	*W*_34_ = 20000

## Appendix A Polarization build-up in the absence of deuteration

One of the basic assumptions in the analysis of data from polarized neutron scattering from dynamically polarized protons in catalase is the existence of a magnetic nuclear spin diffusion barrier. TPP experiments on a small radical molecule in a protiated solvent would not only substantiate this assumption but also provide information on its role in the build-up of proton polarization. It is for this reason that solutions with different concentrations of a chromium(V) complex, abbreviated as EHBA–Cr^V^ (Fig. 17 ▸), in glycerol–water mixtures (1/1 *v*/*v*) have been studied by the method of TPP.

We used three solutions of EHBA–Cr^V^ with concentrations of 5.44 × 10^19^ ml^−1^, 1.53 × 10^19^ ml^−1^ and 0.48 × 10^19^ ml^−1^. As the intensity of the polarization-dependent polarized neutron scattering from dynamically polarized protons is small compared with the strong incoherent scattering from the solvent, *Z*_exp_(*Q*, *t*) was subjected to a two-dimensional Fourier analysis. The Fourier series was terminated at *N*_1_ = 1 and *N*_2_ = 1 in equation (15). The result of the analysis with equations (10)–(12) [Disp-formula fd12] is shown in Fig. 18 ▸(*a*) for the medium concentration of EHBA–Cr^V^. There is a strongly *Q*-dependent change in the intensity with time after each change in the direction of DNP for about 1.5 s. This is interpreted as the period of the build-up of a local proton polarization in R1. After this period, the scattering intensity at different *Q* changes with time at equal rates. This is the signature of time-dependent incoherent scattering. It is intimately related to an accelerated growth of the polarization of the bulk protons shown in Fig. 18 ▸(*b*). The evolution of the proton polarization observed by time-resolved NMR agrees with that of the bulk protons in R2 obtained from the analysis of time-resolved polarized neutron scattering (Fig. 18 ▸ *b*).

The centrepieces of the analysis are the contact term *W*_01_ and the transition probability *W*_12_, which connects the close protons of R1 with those of the bulk in R2. Both *W*_01_ and *W*_12_ increase with increasing concentration of EHBA–Cr^V^. We note that the same variation in *W_ij_* with time has been observed with EHBA–Cr^V^ in deuterated solvents (Stuhrmann, 2015 ▸).

The evolution of the proton polarization is described by two exponentials with characteristic times τ_1_ and τ_2_ (van den Brandt *et al.*, 2006 ▸). With *N*_1_ protons in R1 and the concentration-dependent number *N*_2_ of protons in R2, one obtains

Hence the short time constant τ_1_ increases with the inverse of the concentration of EHBA–Cr^V^. As the mean intermolecular distance *D* (in ångström) = 100/(*c* ^1/3^), with *c* in units of 10^18^ ml^−1^, the short time constant τ_1_ increases with the cube of *d* (Fig. 19 ▸). The characteristic time τ of tyr-369 doped catalase (filled black circle in Fig. 19 ▸) with known inter-radical distances of 20.2, 23.9 and 29.5 Å, does not fall onto the line proposed by the data from EHBA–Cr^V^. A possible response to this apparent discrepancy comes from the distribution of the intermolecular distances between EHBA–Cr^V^ molecules in solution. Molecules at distances smaller than the average distance give rise to a much faster proton polarization. In other words the effective distance, *D*_e_, giving rise to τ is smaller than the average intermolecular distance *D*. An estimate of *D*_e_ is given in the legend of Fig. 19 ▸.

The radius of R1 was determined by trial and error. The best fit of the neutron scattering data is obtained with a radius of 4.2 Å, which is slightly smaller than the radius of the EHBA–Cr^V^ molecule. From this model, it appears that the eight H atoms of the four methylene groups belong to R1, whereas the methyl groups would belong to R2. The other eight hydrogen atoms of R1 would be those of solvent mol­ecules. We cannot exclude the possibility that R1 would include more protons of the solute and less of the solvent.

The choice of a 5 Å radius for R1 in catalase was guided by the results of TPP from free radicals of larger size. Both the octyl derivative of DPPH and the *n*-hexyl derivative of a rodlike biradical molecule show a strong intramolecular proton polarization gradient (Stuhrmann, 2015 ▸). The corresponding radii of R1 are 7 and 5 Å, respectively.

## Appendix B On the time-dependent response of the detector system

Fig. 6 ▸(*a*) shows the variation in the intensity of the primary beam not shielded by the beam stop. Its description by a one-dimensional Fourier series terminated at *N* = 1 reveals a relative change in the intensity of 7 × 10^−4^. A pixelwise analysis of the area of the peaks on the left and right side of the beam stop shows that there is a change in the phase angle φ, defined as φ = arctan(*F*_S_/*F*_C_), of 50° on changing line *Y* of the detector at a constant index of column *X* (Fig. 20 ▸ *a*). Outside the peak area, the regular change in the phase angle φ is lost for two reasons: first, the intensity is too low to allow a precise determination of φ, and second, the time dependence of the scattering from catalase is likely to show up.

The question arises of whether this is a general property of the detector system. The verification of this hypothesis is best done with a time-dependent intensity distribution which is flat and high all over the area detector, as observed with in­coherent neutron scattering from a hydrogenous material. The samples discussed in Appendix *A* [App appa] fulfil this requirement. There is a strong incoherent scattering intensity from the glycerol–water mixture, which is very slightly modulated by a weak time-dependent scattering intensity from EHBA–Cr^V^. Fig. 20 ▸(*b*) shows the phase angles for adjacent lines in two constant columns *X* of the detector. The same phase jump of 50° between neighbouring lines is observed over large stretches of lines *Y*. It may be concluded that the time-dependence of the peak intensity and its propagation into the small-angle region are related to a property of the detector system.

In principle, a two-dimensional phase angle distribution similar to that shown in Fig. 20 ▸(*b*) could be used for a pixelwise correction of the time-dependent scattering intensity *I*(*x*, *y*, *t*) from a proton spin-polarized sample. A suitable reference data set *I*_ref_(*x*, *y*, *t*) would start from time-resolved neutron scattering from clean water, for instance. The very strong, mainly incoherent, scattering intensity would have to be collected over at least 10 000 cycles, as described above. The well resolved two-dimensional phase distribution obtained from *I*_ref_(*x*, *y*, *t*) could allow for a pixelwise correction of the time-dependent scattering (*x*, *y*, *t*) obtained from a dynamically polarized sample.

In practice, the resolution of the two-dimensional phase distribution shown in Fig. 20 ▸(*b*) is hardly suitable for the analysis described above. As for the influence of *I*_ref_(*x*, *y*, *t*) on *Z*_exp_(*Q*, *t*), we assume that the radial average comprising typically 100 pixels will largely wipe out the instrument-dependent time dependence of the scattering intensity *I*_ref_(*x*, *y*, *t*) and that the proton polarization-dependent intensity will prevail in *Z*_exp_(*Q*, *t*).

The intensity of the primary beam leaking through the beam stop shown in Fig. 4 ▸ will, to some extent, spread out along the cathode wires *X* and *Y* which have been hit (Fig. 7 ▸). Along with this goes the time-dependence of the intensity present at the corresponding pixels *x*, *y*, shown in Figs. 6 ▸(*a*) and 20 ▸(*a*).
